# Association between social health status and health-related quality of life among community-dwelling elderly in Zhejiang

**DOI:** 10.1186/s12955-020-01358-4

**Published:** 2020-04-28

**Authors:** Jieming Lu, Zhebin Yu, Xiaocong Zhang, Mengyin Wu, Shujuan Lin, Yao Zhu, Zenghao Xu, Liuqing You, Fang Wei, Mengling Tang, Mingjuan Jin, Jianbing Wang, Kun Chen

**Affiliations:** 1 Department of Epidemiology and Biostatistics, Zhejiang University, School of Medicine, Hangzhou, Zhejiang China; 2 Department of Epidemiology and Biostatistics, Cancer Institute, the Second Affiliated Hospital, Zhejiang University School of Medicine, Hangzhou, China; 3 Department of Epidemiology and Biostatistics, the Children’s Hospital, National Clinical Research Center for Child Health, Zhejiang University School of Medicine, Hangzhou, China

**Keywords:** Social health, Health-related quality of life, Older people

## Abstract

**Background:**

Population aging is an inevitable trend and previous studies have showed the relationship between social health related factors and health-related quality of life (HR-QOL) in the elderly. The objective of this study is to investigate the association of social health status with HR-QOL among community-dwelling elderly in Zhejiang.

**Methods:**

This cross-sectional study was based on community-dwelling elderly individuals from July 2018 to September 2018 in Zhejiang, China. HR-QOL was measured by the 12-item Short-Form Health Survey (SF-12). Social health status was estimated by the long-form of the Social Health Scale for the Elderly (SHSE-L) and classified into three categories (poor, moderate and good). Multivariable linear regression models were conducted to evaluate the association between social health status and HR-QOL (PCS, MCS and SF-12 total score).

**Results:**

A total of 2952 elderly participants were included in this study. The mean age was 70.68 ± 7.75 years (mean ± SD); of the eligible participants, more than half (50.4%) were females; the mean scores were 48.10 ± 8.49, 47.70 ± 7.09 and 47.90 ± 5.86 for PCS, MCS and SF-12 total score, separately. Results from the multivariable models showed that social health status was positively related to HR-QOL after adjusting for covariates. Compared with individuals with a poor social health status, those who had a moderate or good social health status were more likely to report better HR-QOL (for moderate social health status: β = 1.90(95%CI: 1.09, 2.71) for PCS, β = 1.78(1.08, 2.48) for MCS, β = 1.84(1.29, 2.39) for SF-12 total score; for good social health status: β = 3.29(2.24, 4.34) for PCS, β = 3.10(2.12, 4.01) for MCS, β = 3.20(2.48, 3.91) for SF-12 total score).

**Conclusion:**

In our study, we found that social health status was positively associated with HR-QOL among the elderly in Zhejiang. Our findings could provide valuable information for decision-makers to develop interventions to improve the HR-QOL of the elderly.

## Background

With the development of the economy and modern medical techniques, population aging is an inevitable trend. World health organization (WHO) reported that the global population of older people aged 60 years or older reached 0.9 billion in 2015 and may grow further into nearly 2 billion by 2050 [1]. The 2010 census in China showed that individuals aged 60 years or above accounted for 13.3% of the total population in 2010, up by 2.9% as compared with that from the 2000 census [2]. Due to the increasing life expectancy, health-related quality of life (HR-QOL) assessment is considered as a particularly important public health tool for the elderly, which can help determine the burden of preventable disease, injuries, and disabilities [3].

HR-QOL is a common approach to the conceptualization of the broader concept of quality of life (QOL). It has been defined as: “the impact of perceived health on an individual’s ability to live a fulfilling life” [4]. Common dimensions of HR-QOL include physical, psychological, and social components, which may compensate for, or depend on each other. The multidimensionality of HR-QOL makes it pertinent to examine both the respective dimensions of the concept but also the overall score for the population of interest [5]. A number of factors have been identified to be related to HR-QOL among the elderly, such as socioeconomic status, lifestyle behaviors, and health conditions [6–11]. However, social health (such as social support and social relationship) may also affect health-related quality of life in the elderly.

Social health is defined as an ability to accomplish potential and obligations, to manage their life to some extent despite a medical condition, and the ability to participate in social activities including work [12]. Social health contains two aspects: individual and society or a population [13]. Social health of an individual is usually explained as “well-being”, “adjustment” or other terms rather than health [14], and it can be measured from two aspects: social support (SS) and social adjustment (SA). Social health for a society mainly reflects the neighborhood environment [15]. A number of studies have focused on the association between a single level of social health and HR-QOL among the elderly [16–19]. A study conducted in China found the relationship between social support and HR-QOL, mediating role of resilience [20]; A longitudinal study indicated that consistent participation in religious activities, friendship organizations, leisure/culture clubs, family/school reunion, and volunteer work could improve the quality of life among middle-aged and older Koreans [21]; And a cross-sectional study in Netherlands showed that multiple environmental factors were associated with quality of life in the elderly [22]. However, up to now, limited studies can be available for evaluating the associations of individual and society levels of social health with health-related quality of life in the elderly.

Nowadays, a comprehensive structured scale called the Social Health Scale for the Elderly (SHSE) has been developed to fill the gap in social health status measurement [15]. Herein, we reported a cross-sectional study to explore the relationship between social health status and HR-QOL among the elderly in Zhejiang, China.

## Method

### Study design and study participants

Our data were derived from “Development of health assessment instruments and parameter specification for the elderly” project, which has been described in detail previously [15]. Briefly, this was a cross-sectional study based on community-dwelling elderly individuals from July 2018 to September 2018 in Zhejiang, China. Subjects were enrolled from 12 communities of Yiwu county in Jinhua city and Jianggan district in Hangzhou city, Zhejiang. A convenience sampling method stratified by gender and age was used in each community, based on the distribution of age and gender in the elderly population in Zhejiang. Community-dwelling persons aged 60 years and older were recruited in the current study. Individuals were not eligible if they were bed-ridden or could not participate in their daily living activities by themselves due to serious physical disease or had cognitive problems. All participants were informed to be face-to-face interviewed at adjacent community health centers by strictly trained interviewers. This study was approved by the Ethics Committee of School of Medicine, Zhejiang University, Hangzhou, China. Written informed consent was obtained from all participants before the face-to-face interview.

### Measurement of social health status

Social health status was measured through the long-form of the SHSE (SHSE-L). The 25-item scale was designed to assess social health status of the elderly (including two aspects: individual and society). Individual dimensions consist of Social Support (including emotional support, information support, and instrumental support), and Social Adjustment (including social participation, social relationships, and ego system); Social dimension is perceived environment resource (including built environment and community manage/service) [15]. Three different scoring methods were used in this study: 1. For multiple option items, scoring was based on the number of selected options; 2. For single option items, scoring was based on the serial number of the single option; 3. For items about passage time, scoring was based on the estimated time and means of transportation. Detailed information and scoring criteria could be available in the additional file 1. Crude total score of scale was transformed into standard score (T score), based on a previous study performed by Hembling (1984) [23]. T score was divided into three categories of social health status according to standard norm: poor (T score ≤ 40), moderate(40 < T score ≤ 60), and good (60 < T score ≤ 90) [15]. The SHSE-L has been established and validated in the Hangzhou elderly population. The test-retest variability was 0.77, internal consistency reliability- Cronbach’s alpha was 0.79, concurrent validity was 0.64, and goodness of fit was 0.95 in construct validity [15].

### Measurement of HR-QOL

We used the 12-item Short-Form Health Survey (SF-12) to evaluate the HR-QOL; SF-12 is a concise questionnaire that evaluates the quality of life in Chinese elderly [24]. The 12 items in SF-12 are grouped into 8 main domains: general health, physical functioning, physical role functioning, bodily pain, vitality, social functioning, emotional role functioning, and mental health. These dimensions can be categorized into the Physical Component Summary (PCS) and the Mental Component Summary (MCS). The PCS was calculated based on the sum of the first four items, and the MCS was determined based on the sum of the last four items [25]. We calculated the scores of PCS, MCS, and SF-12 total score that represented a mean value of the PCS and MCS scores. The detailed calculations of PCS and MCS have been published previously [26], and total score of SF-12 was calculated based on the measurement of SF-36 total score in previous studies [26–28].

### Assessment of covariates

Demographic data were obtained using a standard questionnaire. Three categories of covariates were included in the analyses: socio-demographic characteristics, health-related behaviors, and health conditions. Socio-demographic characteristics included the following variables: region (urban or rural), gender (male or female), age group(60–74 or ≥ 75 years), marital status (married, widowed or others), education level (lower than primary school, primary school, middle school, or high school and higher), monthly income(< 1000, 1000–1999, 2000–2999,3000–3999,≥4000 Chinese Yuan (CNY)), living arrangement (living with spouse, living with children, living with spouse and children, living alone or others), and body mass index (BMI). BMI was calculated as weight in kilograms divided by the square of height in meters and divided into three levels according to the Chinese BMI criteria:<18.5,18.5–23.9 or ≥ 24.0 kg/m^2^. Health-related behaviors included smoking (yes or no), drinking alcohol (yes or no), drinking tea (yes or no) and weekly physical activity (≤1 time, 2–4 times or>4 times). Smokers were defined as individuals who smoked 1 cigarette or more per day for 3 months or more. Alcohol drinkers were defined as individuals who drank one glass of beer, liquor, yellow rice wine or red wine per week for more than 1 year [29]. Tea consumption was defined as drinking at least one cup of tea per day for 1 year. Valid physical activity was defined as at least 15 min at a time [30]. Health conditions included depression symptom (yes or no) and the number of chronic conditions (none, one, two or more). Depression symptom was measured by the Geriatric Depression Scale (GDS). Chronic conditions were self-reported, including hypertension, coronary heart disease (CHD), diabetes, stroke, osteoporosis, and arthritis.

### Statistical analysis

Continuous and categorical variables were described as mean ± standard deviation (SD) and *n* (%), respectively. T-test and Analysis of Variance (ANOVA) were used to compare the differences in different categories for continuous variables of PCS, MCS and SF-12 total score. We used multivariate linear regression models to estimate beta (β) and 95% confidence intervals (95% CI) for examining the associations of social health status with PCS or MCS or SF-12 total score. All models were adjusted for potential covariates. Collinearity was assessed by using the correlation matrix of the estimated parameters, and variance inflation factor (VIF). For all analyses, *P*-value < 0.05 (two-sided) was considered to be statistically significant. All data were analyzed using the Statistical Package for Social Sciences (SPSS) version 24.0.

## Results

A total of 3161 elderly participants were recruited in our study, and we excluded 15 participants with incorrect information, 81 participants aged < 60 years and 113 participants with incomplete information of SF-12, and a total of 2952 participants were included in the final analysis. The number of poor, moderate and good social health status was 476, 1994, and 482, respectively. The mean scores were 48.10 ± 8.49(mean ± SD), 47.70 ± 7.09 and 47.90 ± 5.86 for PCS, MCS and SF-12 total score, separately. Mean age for all participants was 70.68 ± 7.75 years old, and 1487(50.4%) were females. Approximately 20% were not married, and less than 20% had no educational experience. Nearly 50% of participants lived in the urban regions and half of the participants had less than 2000 yuan per month of income. About 54.4% of elderly people lived with a spouse, and 49.4% had a normal BMI (Table 1). A significant difference in PCS was observed across the groups based on social health, gender, age, marital status, education level, monthly income, living arrangement, BMI, smoking, alcohol consumption, tea consumption, weekly physical activity, depression symptom and number of chronic conditions (*P* < 0.01). Similar results were observed for MCS, except for gender, age, smoking, alcohol consumption and number of chronic conditions. For SF-12 total score, there was a significant difference across the groups of all demographic characteristics (*P* < 0.01) (Table 1).

**Table 1:** HR-QOL according to different groups of demographic characteristics in the Zhejiang elderly

Variables	Overall(*n* = 2952)	HR-QOL					
		PCS	*P* value	MCS	*P* value	SF-12 total score	*P* value
Social health status			< 0.01		< 0.01		< 0.01
Poor	476(16.12)	45.07 ± 9.64		45.61 ± 8.23		45.34 ± 6.68	
Moderate	1994(67.55)	48.25 ± 8.34		47.80 ± 6.96		48.02 ± 5.73	
Good	482(16.33)	50.45 ± 6.89		49.36 ± 5.30		49.91 ± 4.50	
Region			0.18		< 0.01		< 0.01
Rural	1518(51.42)	47.89 ± 8.20		46.10 ± 6.85		47.00 ± 5.96	
Urban	1434(48.58)	48.31 ± 8.78		49.39 ± 6.95		48.85 ± 5.60	
Gender			< 0.01		> 0.05		< 0.01
Male	1464(49.61)	48.94 ± 8.35		47.96 ± 6.70		48.45 ± 5.67	
Female	1487(50.39)	47.26 ± 8.54		47.45 ± 7.45		47.35 ± 6.00	
Age group (Years)			< 0.01		0.33		< 0.01
60–74	2069(70.09)	49.49 ± 7.80		47.78 ± 6.73		48.64 ± 5.40	
≥75	883(29.91)	44.84 ± 9.12		47.51 ± 7.88		46.17 ± 6.50	
Marital status			< 0.01		< 0.05		< 0.01
Married	2370(80.28)	48.65 ± 8.27		47.87 ± 6.91		48.26 ± 5.74	
Widowed	491(16.63)	45.33 ± 9.14		47.10 ± 7.86		46.21 ± 6.17	
Others	91(3.08)	48.44 ± 7.65		46.50 ± 7.17		47.48 ± 5.70	
Education level			< 0.01		< 0.01		< 0.01
Lower than primary school	556(19.01)	45.94 ± 8.68		46.41 ± 7.50		46.18 ± 6.20	
Primary school	953(32.58)	47.78 ± 8.57		46.76 ± 7.03		47.28 ± 6.01	
Middle school	855(29.23)	49.37 ± 8.07		48.56 ± 6.81		48.97 ± 5.40	
High school or higher	567(19.38)	48.87 ± 8.38		49.23 ± 6.78		49.05 ± 5.40	
Monthly income (CNY)			< 0.01		< 0.01		< 0.01
<1000	722(25.38)	47.247 ± 8.43		45.74 ± 7.50		46.50 ± 6.32	
1000–1999	706(24.82)	48.05 ± 8.45		47.20 ± 6.78		47.62 ± 6.04	
2000–2999	570(20.04)	48.49 ± 8.29		47.87 ± 7.48		48.18 ± 5.71	
3000–3999	388(13.64)	47.86 ± 9.28		49.44 ± 6.34		48.65 ± 5.45	
≥4000	459(16.13)	49.31 ± 8.31		50.35 ± 5.99		49.83 ± 4.75	
Living arrangement			< 0.01		< 0.01		< 0.01
Live with spouse	1607(54.44)	48.71 ± 8.20		48.06 ± 6.69		48.39 ± 5.62	
Live with children	296(10.03)	45.70 ± 9.01		46.69 ± 7.97		46.19 ± 6.20	
Live with spouse and children	573(19.41)	49.02 ± 8.21		48.03 ± 7.17		48.52 ± 5.69	
Live alone	322(10.91)	45.65 ± 8.96		46.99 ± 7.39		46.32 ± 6.25	
Others	154(5.22)	47.95 ± 8.79		46.16 ± 7.94		47.05 ± 6.21	
BMI (kg/m^2^)			< 0.01		< 0.01		< 0.01
<18.5	184(6.44)	45.04 ± 9.21		46.81 ± 8.71		45.93 ± 6.66	
18.5–23.9	1412(49.42)	48.47 ± 8.40		47.38 ± 7.24		47.93 ± 5.87	
≥24.0	1261(44.14)	48.08 ± 8.41		48.21 ± 6.53		48.15 ± 5.62	
Smoking			< 0.01		0.81		< 0.01
Yes	388(13.14)	49.73 ± 8.42		47.62 ± 6.62		48.68 ± 5.76	
No	2543(86.14)	47.84 ± 8.48		47.71 ± 7.17		47.78 ± 5.87	
Alcohol drinking			< 0.01		0.33		< 0.01
Yes	558(19.04)	49.95 ± 7.73		47.96 ± 6.70		48.96 ± 5.40	
No	2373(80.96)	47.65 ± 8.60		47.64 ± 7.19		47.65 ± 5.94	
Tea drinking			< 0.01		< 0.01		< 0.01
Yes	964(32.89)	49.28 ± 8.22		48.52 ± 7.01		48.90 ± 5.66	
No	1967(67.11)	47.51 ± 8.57		47.30 ± 7.10		47.41 ± 5.90	
Weekly physical activity			< 0.01		< 0.01		< 0.01
≤1 time	1064(37.35)	46.06 ± 9.26		46.39 ± 7.62		46.23 ± 6.47	
2–4 times	1524(53.49)	49.24 ± 7.69		48.90 ± 6.54		49.07 ± 5.08	
> 4 times	261(9.16)	50.23 ± 8.00		47.39 ± 6.43		48.81 ± 5.49	
Depression symptom			< 0.01		< 0.01		< 0.01
Yes	767(26.19)	49.09 ± 7.91		48.51 ± 6.25		48.80 ± 5.17	
No	2162(73.81)	45.43 ± 9.35		45.53 ± 8.66		45.48 ± 6.91	
Number of chronic conditions			< 0.01		0.15		< 0.01
0	777(26.32)	50.56 ± 7.15		47.75 ± 6.29		49.15 ± 5.01	
1	1184(40.11)	49.00 ± 7.84		47.41 ± 7.23		48.21 ± 5.77	
≥2	991(33.57)	45.08 ± 9.30		48.01 ± 7.50		46.54 ± 6.30	

Table 2 summarizes the adjusted association of social health status with HR-QOL. As compared with individuals with a poor social health status, subjects who had a moderate or good social health status were more likely to report better HR-QOL (for moderate social health status: β = 1.90(95%CI:1.09, 2.71) for PCS; β = 1.78(1.08, 2.48) for MCS; β = 1.84(1.29, 2.39) for SF-12 total score; for good social health status: β = 3.29(2.24, 4.34) for PCS; β = 3.10(2.20, 4.01) for MCS; β =3.20(2.48, 3.91) for SF-12 total score). No collinearity was observed among the variables included in these models.

**Table 2:** Beta values and 95% confidence intervals of HR-QOL by social health status in the Zhejiang elderly

Social health status	PCS	MCS	SF-12 total score
	β(95%CI)	β(95%CI)	β(95%CI)
Poor	Ref.	Ref.	Ref.
Moderate	1.90(1.09,2.71)	1.78(1.08,2.48)	1.84(1.29,2.39)
Good	3.29(2.24,4.34)	3.10(2.20,4.01)	3.20(2.48,3.91)

Adjusted for Region, Gender, Age group, Marital status, Education level, Monthly income (CNY), Living arrangement, Smoking, Alcohol drinking, Tea drinking, Depression symptom, Weekly physical activity, BMI level, Number of chronic conditions

As for covariates, individuals who were 75 years and older, or had a BMI < 18.5 kg/m^2^ or chronic conditions, or did physical activity less than 2 times weekly, were more likely to report lower PCS. Participants who lived in urban regions and lived with spouse and children, or had a monthly income over 3000 Yuan or BMI ≥ 24 kg/m^2^ or no depression symptom, were more likely to report higher MCS. As for SF-12 total score, subjects who lived in the rural regions, were female or 75 years and older or non-tea drinkers, whose monthly income less than 4000 yuan and BMI < 18.5 kg/m^2^, or had chronic conditions with depression symptom were more likely to report lower SF-12 total score **(Supplementary Tables S****1****–****3**).

## Discussion

This present study aimed at assessing whether social health status was associated with HR-QOL in the Zhejiang elderly. Compared with the elderly with a poor social health status, those who had a better social health status were more likely to report higher scores of PCS, MCS and SF-12 total score, after adjusting for potential covariates. These findings suggest that social health status may be considered as a comprehensive indicator of HR-QOL and may help to develop interventions to improve the quality of life in the elderly.

In this study, social health status was found to be positively associated with PCS among the elderly, which was supported by previous studies for the association of social health related factors with physical health among the elderly [31–35]. A cross-sectional study conducted in Kuwaitis showed that having children, perception of social support, frequency of contact with kin, and strength of relationships with kin were important modulators of somatic symptoms among the elderly [36]. And a longitudinal study in Japan suggested that interaction between environment and multifaceted social relationships had the strongest impact on functional ability for the elderly [37]. Increased social relationships had beneficial effects in fostering the elders’ physical and cognitive functions through active participation in social activities and building social networks [38].

Meanwhile, social health status was a positive factor of MCS, which was supported by previous studies for the association of social health related factors with mental health among the elderly [39–43]. The wave three of Nord-Trøndelag Health Study (HUNT3 Study) indicated that lesser psychological distress in the elderly was dependent on better scores on social support [44]; An observational study demonstrated that in the healthy elderly, participating in a social activity could help improve psychological distress [45]. Depression was one of the most prevalent mental disorders in the elderly population and was associated with risk of disability and mortality [46], and several studies have suggested the associations of social support, social participation, and social relationships with depression symptom [47–49].

Perceived environment resource is also an important dimension of social health. The World Report on Aging and Health recommends that decision-makers need to build supportive and enabling environments, which can help people build and maintain capacity (for example, a walkable environment may foster physical activity) [50]. A study in Hong Kong showed that environmental walkability was associated with HR-QOL among older adults [51]. Moreover, some perceived environment resources, such as safety from traffic and street noise were associated with HR-QOL [16]. The changes in the environment around the community may also affect health-related quality of life in the elderly.

Additionally, we noticed that participants who lived in the rural region, were females, aged 75 years and older, were not tea drinkers, or had less than 4000 yuan of monthly income, BMI < 18.5 kg/m^2^, or chronic conditions with depression symptom were more likely to report lower SF-12 total score. Also, we noticed that monthly income over 3000 CNY was a positive factor of MCS, which was not comparable with a previous study [10]. Possible explanations could be: most of older people in this study were retirees and got money from their children or pension. Consequently, those older people who got more money per month could have less economic hardship and a peaceful life attitude.

Our study had some important strengths. It was the first study that explored the influential factors of HR-QOL among the elderly from a perspective of social health status. Moreover, we enrolled a total of 2952 elderly people, which could be considered as a large sample compared with similar studies. Finally, the combination of indicators (PCS, MCS, and SF-12 total score) could improve the meaning of our results. However, our study also had several limitations. Firstly, our study was a cross-sectional study and the causal relationship could not be demonstrated. Secondly, selection bias might not be avoided due to the nonrandomized sampling and relatively low response rate. However, the age and sex distributions of the study population were similar to the Zhejiang elderly population. Finally, although we adjusted for many possible confounders (such as smoking, alcohol drinking, and tea consumption), we lacked information on other potential confounders, such as dietary patterns.

## Conclusion

In summary, to our knowledge, this was the first study to explore the relationship between social health status and HR-QOL among Chinese elderly people. Our results showed that social health status was positively associated with HR-QOL among the elderly in Zhejiang, after adjusting for potential confounding factors. These findings could provide an understanding of how social health status affect HR-QOL of older adults, as well as a new insight into a reference for promoting the HR-QOL among Chinese elderly people.

## Supplementary information

**Additional file 1.**

**Additional file 2: TableS1.** The multiple linear regression results of PCS among the elderly. **TableS2.** The multiple linear regression results of MCS among the elderly. **TableS3.** The multiple linear regression results of SF-12 total score among the elderly.

## Data Availability

The datasets generated during and/or analyzed during the current study are not publicly available, but are available from the corresponding author who was an organizer of the study.

## Abbreviations

HR-QOL: Health-related quality of life
SF-12: The 12-item Short-Form Health Survey
SHSE-L: The long-form of the Social Health Scale for the Elderly
PCS: Physical component summary
MCS: Mental component summary
WHO: World health organization
CDC: Centers for Disease Control and Prevention
QoL: Quality of life
SP: Social participation
SS: Social adjustment
BMI: Body mass index
CNY: Chinese yuan
CHD: Coronary heart disease.
GDS: Geriatric Depression Scale
SD: Standard deviation
ANOVA: Analysis of Variance
CI: Confidence interval
VIF: Variance inflation factor
SPSS: Statistical Package for Social Sciences
