# Supplementary material for: Association between social health status and health-related quality of life among community-dwelling elderly in Zhejiang
Source: Health Qual Life Outcomes. 2020 Apr 28;18:110. doi: 10.1186/s12955-020-01358-4 (PMC7189640; doi:10.1186/s12955-020-01358-4)
Supplement: Supplementary file 1 — Additional file 1. [file 12955_2020_1358_MOESM1_ESM.docx]

**The Social Health Scale for the Elderly(long form)**

**The options of question D01-D12:**

[1] = None

[2] = Children (natural/adopted children);

[3] = Partner (spouse/a person of the opposite sex in a permanent relationship);

[4] = Friends;

[5] = Other relatives (blood relatives or close relatives by marriage excluding natural children and spouse)

[6] = Others

***Question B01-B15 can have multiple answers. Please draw a circle on the option.***

**D01** Who can be a listener when you want to pour out your heart? [1] [2] [3] [4] [5] [6]

**D02** Who accepts you totally, including your worst points? [1] [2] [3] [4] [5] [6]

**D03** Who can understand your problems/ideas? [1] [2] [3] [4] [5] [6]

**D04** Who can support you in major decisions you make? [1] [2] [3] [4] [5] [6]

**D05** Who can care about you, regardless of what is happening to you? [1] [2] [3] [4] [5] [6]

**D06** Who can accompany you to pass the time? [1] [2] [3] [4] [5] [6]

**D07** Who can comfort you when you need it? [1] [2] [3] [4] [5] [6]

**D08** Who can give you information to help you understand a situation? [1] [2] [3] [4] [5] [6]

**D09** Who can tell you where you need to improve in a thoughtful manner? [1] [2] [3] [4] [5] [6]

**D10** Who can give you useful suggestions that help you to make decisions? [1] [2] [3] [4] [5] [6]

**D11** Who can help you with daily chores if you were sick? [1] [2] [3] [4] [5] [6]

**D12** Who can give you financial aid? [1] [2] [3] [4] [5] [6]

**The options of question D13-D17: *single answers. Please draw a circle on the option.***

[1] = Less than once every three months;

[2] = Once/more than once every three months, but less than once every month;

[3] = One to four times every month;

[4] = Two to four times every week;

[5] = More than four times every week;

**D13** How often did you do housework in the last year? [1] [2] [3] [4] [5]

**D14** How often did you participate in collective recreational activities in the last year? [1] [2] [3] [4] [5]

**D15**How often did you chat with children for a long time in the last year? [1] [2] [3] [4] [5]

**D16** How often did you chat with friends for a long time in the last year? [1] [2] [3] [4] [5]

**D17**How often did you chat with other relatives in the last year? [1] [2] [3] [4] [5]

**The options of question D18: *single answers. Please draw a circle on the option.***

[1] = Almost never

[2] = Once in a while

[3] = About half the time

[4] = Most of the time

[5] = Always

**D18** How much spare time have you spent on hobbies or interests in the last year? [1] [2] [3] [4] [5]

***Question D19 can have multiple answers. Please draw a circle on the option.***

**D19** Which of the following manufactured landscapes have been seen in/around the community you lived in the last year?

[1] None [2] Artificial hill [3] Artificial lake and pond

[4] Artificial lawn [5] Garden building [6] Artificial planting flowers and trees

***QuestionD20-D22 have single answers. Please write the answer on the line.***

**D20** If you go to the nearest public transit facility (bus stop/subway station/bicycle rental station) from your house by , you should spend minutes.

[1] Walk [2] Bicycle [3] Electric bicycle [4] Bus [5] Private car

**D21** If you go to the nearest fitness/recreation facility (outdoor exercise machine/gymnasium/university for the elderly/community entertainment room) from your house by , you should spend minutes.

[1] Walk [2] Bicycle [3] Electric bicycle [4] Bus [5] Private car

**D22** If you go to the nearest medical institution (general hospital/special hospital/community health service center /township hospital/village clinic/private clinic) from your house by , you should spend minutes.

[1] Walk [2] Bicycle [3] Electric bicycle [4] Bus [5] Private car

***Question D23 can have multiple answers. Please draw a circle on the option.***

**D23**Which of the following public facilities have been seen in/around the community you lived in the last year?

[1] None [2] Footpath [3] Bicycle lane [4] Special entrance for the disabled [5] Free parking lot

[6] Free playground/garden [7] Free swimming pool [8] Free stadium [9] Dumping site

***Question D24 has single answer. Please draw a circle on the option.***

**D24** How many times has the community you lived in organized the activities in the last year?

[1] Never [2] Once or twice [3] Three or four times [4] Five or six times [5] More than six times

***Question D25 can have multiple answers. Please draw a circle on the option.***

**D25**Which of the following free public services have been offered by the community you lived in in the last year?

[1] None

[2] Door-to-door medical service

[3] Chatting with the oldest old, the disabled/ bedridden/solitary elderly

[4] Handling the dispute between neighbors

[5] Health education

[6] Legal aid/advice

[7] Distributing supply

[8] Nursing the oldest old, the disabled/ bedridden/solitary elderly

**The scoring method of items in the Social Health Scale for the Elderly (draft)**

| Variables | Recording rule^#^ | | | | |
| --- | --- | --- | --- | --- | --- |
| D01-D12, D19 | [1] = 1 | 1 = 2 | 2 = 3 | 3 = 4 | 4 or more= 5 |
| D13-D18, D24 | [1] = 1 | [2] = 2 | [3] = 3 | [4] = 4 | [5] = 5 |
| D20-D22^*^ | 31^a^ or more = 1^b^ | 21^a^ to 30^a^ = 2 | 11^a^ to 20^a^ = 3 | 6^a^ to 10^a^ = 4 | 5^a^ or less = 5 |
| D23, D25 | [1] = 1 | 1, 2 = 2 | 3, 4 = 3 | 5, 6 = 4 | 7 or higher = 5 |
| Note  ^#^ The left of equal sign: the number of selected option excluding “none” (Arabic numerals)/option (parenthesized Arabic numerals)/calculation (superscripted Arabic numerals). The right of equal sign means the raw score of each variable.  ^*^ Speed conversion equation (parenthesized Arabic numerals mean options): [2] = [1]*3, [3] = [1]*6, [4] = [1]*6, [5] = [1]*12.  ^a^ Calculation: the length of walking time (minutes) after converting based on the speed conversion equation.  ^b^ If the length of time cannot been estimated because the interviewee has never gone to such site, then this score will be chosen. | | | | | |
