# Supplementary material for: Association between social health status and health-related quality of life among community-dwelling elderly in Zhejiang
Source: Health Qual Life Outcomes. 2020 Apr 28;18:110. doi: 10.1186/s12955-020-01358-4 (PMC7189640; doi:10.1186/s12955-020-01358-4)
Supplement: Supplementary file 2 — Additional file 2: TableS1. The multiple linear regression results of PCS among the elderly. TableS2. The multiple linear regression results of MCS among the elderly. TableS3. The multiple linear regression results of SF-12 total score among the elderly. [file 12955_2020_1358_MOESM2_ESM.docx]

Supplementary Table

TableS1. The multiple linear regression results of PCS among the elderly

|  | | | |
| --- | --- | --- | --- |
| independent variables | β | 95% CI | |
|  |  | Lower | Upper |
| Social health status |  |  |  |
| poor | Ref | | |
| moderate | 1.90** | 1.09 | 2.71 |
| good | 3.29** | 2.24 | 4.34 |
| Region | 0.10 | -0.78 | 0.98 |
| Gender | -0.60 | -1.29 | 0.10 |
| Age group | -3.25** | -3.94 | -2.56 |
| Marital status |  |  |  |
| married | Ref | | |
| widowed | 0.72 | -0.60 | 2.03 |
| others | 0.47 | -1.31 | 2.24 |
| Education level |  |  |  |
| Never go to school | Ref | | |
| Primary school | 0.04 | -0.83 | 0.92 |
| Middle school | 0.57 | -0.19 | 1.34 |
| High school or higher | 0.51 | -0.44 | 1.45 |
| Monthly income |  |  |  |
| ＜1000 | Ref | | |
| 1000-1999 | -0.52 | -1.34 | 0.30 |
| 2000-2999 | 0.41 | -0.56 | 1.37 |
| 3000-3999 | -0.32 | -1.44 | 0.81 |
| ≥4000 | 0.36 | -0.79 | 1.51 |
| Living arrangement |  |  |  |
| Live with spouse | -0.05 | -1.40 | 1.31 |
| Live with children | -1.88** | -3.51 | -0.26 |
| Live with spouse and children | -0.13 | -1.59 | 1.32 |
| Live alone | -1.17 | -2.84 | 0.50 |
| other | Ref | | |
| BMI level |  |  |  |
| 18.5-23.9 | Ref | | |
| ＜18.5 | -2.86** | -4.06 | -1.67 |
| ≥24 | -0.14 | -0.73 | 0.45 |
| Smoking | -0.08 | -0.87 | 1.02 |
| Alcohol drinking | 0.94* | 0.14 | 1.74 |
| Tea drinking | 1.28* | 0.61 | 1.95 |
| Weekly Physical activity |  |  |  |
| ≤1 time | Ref | | |
| 2-4 times | 1.84** | 1.15 | 2.52 |
| 4 times | 1.41** | 0.86 | 1.95 |
| Depression symptom | -2.22** | -2.88 | -1.55 |
| Number of chronic conditions |  |  |  |
| 0 | Ref | | |
| 1 | -1.26** | -1.98 | -0.55 |
| ≥2 | -5.01** | -5.78 | -4.24 |
| *P<0.05 |  |  |  |
| **P<0.01 |  |  |  |

TableS2. The multiple linear regression results of MCS among the elderly

|  | | | |
| --- | --- | --- | --- |
| independent variables | β | 95% CI | |
|  |  | Lower | Upper |
| Social health status |  |  |  |
| poor | Ref | | |
| moderate | 1.78** | 1.08 | 2.48 |
| good | 3.10** | 2.20 | 4.01 |
| Region | 2.79** | 2.03 | 3.55 |
| Gender | -0.40 | -1.00 | 0.20 |
| Age group | 0.51 | -0.09 | 1.11 |
| Marital status |  |  |  |
| married | Ref | | |
| widowed | 1.35* | 0.21 | 2.48 |
| others | 0.24 | -1.29 | 1.77 |
| Education level |  |  |  |
| Never go to school | Ref | | |
| Primary school | 0.08 | -0.67 | 0.83 |
| Middle school | 0.23 | -0.44 | 0.89 |
| High school or higher | -0.06 | -0.87 | 0.76 |
| Monthly income |  |  |  |
| ＜1000 | Ref | | |
| 1000-1999 | 0.69 | -0.02 | 1.39 |
| 2000-2999 | 0.52 | -0.31 | 1.35 |
| 3000-3999 | 1.72** | 0.75 | 2.69 |
| ≥4000 | 2.21** | 1.22 | 3.20 |
| Living arrangement |  |  |  |
| Live with spouse | 1.15 | -0.02 | 2.33 |
| Live with children | -0.24 | -1.64 | 1.17 |
| Live with spouse and children | 1.45* | 0.19 | 2.71 |
| Live alone | 0.17 | -1.28 | 1.61 |
| other | Ref | | |
| BMI level |  |  |  |
| 18.5-23.9 | Ref | | |
| ＜18.5 | -0.83 | -1.87 | 0.20 |
| ≥24 | 0.84* | 0.32 | 1.35 |
| Smoking | -0.51 | -1.33 | 0.31 |
| Alcohol drinking | -0.19 | -0.88 | 0.50 |
| Tea drinking | -0.30 | -0.88 | 0.28 |
| Weekly Physical activity |  |  |  |
| ≤1 time | Ref | | |
| 2-4 times | 0.50 | -0.09 | 1.09 |
| 4 times | 0.56 | 0.09 | 1.03 |
| Depression symptom | -2.93** | -3.51 | -2.36 |
| Number of chronic conditions |  |  |  |
| 0 | Ref | | |
| 1 | -0.52 | -1.14 | 0.10 |
| ≥2 | -0.50 | -1.17 | 0.16 |
| *P<0.05 |  |  |  |
| **P<0.01 |  |  |  |

TableS3. The multiple linear regression results of SF-12 total score among the elderly

|  | | | |
| --- | --- | --- | --- |
| independent variables | β | 95% CI | |
|  |  | Lower | Upper |
| Social health status |  |  |  |
| poor | Ref | | |
| moderate | 1.84** | 1.29 | 2.39 |
| good | 3.20** | 2.48 | 3.91 |
| Region | 1.45** | 0.85 | 2.04 |
| Gender | -0.50* | -0.97 | -0.03 |
| Age group | -1.37** | -1.84 | -0.90 |
| Marital status |  |  |  |
| married | Ref | | |
| widowed | 1.03* | 0.14 | 1.93 |
| others | 0.35 | -0.85 | 1.56 |
| Education level |  |  |  |
| Never go to school | Ref | | |
| Primary school | 0.06 | -0.53 | 0.65 |
| Middle school | 0.40 | -0.12 | 0.92 |
| High school or higher | 0.22 | -0.42 | 0.87 |
| Monthly income |  |  |  |
| ＜1000 | Ref | | |
| 1000-1999 | 0.08 | -0.47 | 0.64 |
| 2000-2999 | 0.46 | -0.19 | 1.12 |
| 3000-3999 | 0.70 | -0.06 | 1.47 |
| ≥4000 | 1.28** | 0.50 | 2.06 |
| Living arrangement |  |  |  |
| Live with spouse | 0.55 | -0.37 | 1.48 |
| Live with children | -1.06 | -2.16 | 0.05 |
| Live with spouse and children | 0.66 | -0.33 | 1.65 |
| Live alone | -0.50 | -1.64 | 0.64 |
| other | Ref | | |
| BMI level |  |  |  |
| 18.5-23.9 | Ref | | |
| ＜18.5 | -1.85** | -2.66 | -1.04 |
| ≥24 | 0.34 | -0.05 | 0.75 |
| Smoking | -0.29 | -0.94 | 0.35 |
| Alcohol drinking | 0.38 | -0.17 | 0.92 |
| Tea drinking | 0.49* | 0.03 | 0.94 |
| Weekly Physical activity |  |  |  |
| ≤1 time | Ref | | |
| 2-4 times | 1.17 | 0.70 | 1.63 |
| 4 times | 0.98 | 0.61 | 1.36 |
| Depression symptom | -2.57** | -3.03 | -2.12 |
| Number of chronic conditions |  |  |  |
| 0 | Ref | | |
| 1 | -0.89** | -1.38 | -0.41 |
| ≥2 | -2.76** | -3.28 | -2.23 |
| *P<0.05 |  |  |  |
| **P<0.01 |  |  |  |
